# Complete genome sequence of *Haloterrigena turkmenica* type strain (4k^T^)

**DOI:** 10.4056/sigs.681272

**Published:** 2010-02-28

**Authors:** Elisabeth Saunders, Brian J. Tindall, Regine Fähnrich, Alla Lapidus, Alex Copeland, Tijana Glavina Del Rio, Susan Lucas, Feng Chen, Hope Tice, Jan-Fang Cheng, Cliff Han, John C. Detter, David Bruce, Lynne Goodwin, Patrick Chain, Sam Pitluck, Amrita Pati, Natalia Ivanova, Konstantinos Mavromatis, Amy Chen, Krishna Palaniappan, Miriam Land, Loren Hauser, Yun-Juan Chang, Cynthia D. Jeffries, Thomas Brettin, Manfred Rohde, Markus Göker, James Bristow, Jonathan A. Eisen, Victor Markowitz, Philip Hugenholtz, Hans-Peter Klenk, Nikos C. Kyrpides

**Affiliations:** 1 DOE Joint Genome Institute, Walnut Creek, California, USA; 2 Los Alamos National Laboratory, Bioscience Division, Los Alamos, New Mexico, USA; 3 DSMZ – German Collection of Microorganisms and Cell Cultures GmbH, Braunschweig, Germany; 4 Biological Data Management and Technology Center, Lawrence Berkeley National Laboratory, Berkeley, California, USA; 5 Oak Ridge National Laboratory, Oak Ridge, Tennessee, USA; 6 HZI – Helmholtz Centre for Infection Research, Braunschweig, Germany; 7 University of California Davis Genome Center, Davis, California, USA

**Keywords:** extreme halophile, thermophile, free-living, aerobic, non-pathogenic, carotenoids-containing, *Halobacteriaceae*, GEBA

## Abstract

*Haloterrigena turkmenica* (Zvyagintseva and Tarasov 1987) Ventosa *et al*. 1999, comb. nov. is the type species of the genus *Haloterrigena* in the euryarchaeal family *Halobacteriaceae*. It is of phylogenetic interest because of the yet unclear position of the genera *Haloterrigena* and *Natrinema* within the *Halobacteriaceae*, which created some taxonomic problems historically. *H. turkmenica*, was isolated from sulfate saline soil in Turkmenistan, is a relatively fast growing, chemoorganotrophic, carotenoid-containing, extreme halophile, requiring at least 2 M NaCl for growth. Here we describe the features of this organism, together with the complete genome sequence, and annotation. This is the first complete genome sequence of the genus *Haloterrigena*, but the eighth genome sequence from a member of the family *Halobacteriaceae*. The 5,440,782 bp genome (including six plasmids) with its 5,287 protein-coding and 63 RNA genes is part of the *** G****enomic* *** E****ncyclopedia of* *** B****acteria and* *** A****rchaea * project.

## Introduction

Strain 4k^T^ (= DSM 5511 = ATCC 51198 = VKM B-1734) is the type strain of the species *Haloterrigena turkmenica*, which is the type species of the genus *Haloterrigena* [1,2]. The strain was initially described in 1987 as *Halococcus turkmenicus* VKM B-1734 (basonym) by Zvyagintseva and Tarasov [3]. In 1999, Ventosa *et al.* proposed to transfer *H. turkmenicus* 4k as the type strain of the species *H. turkmenica* to the new genus *Haloterrigena* [1], whose name means salt, *halos,* (-requiring) and born from the earth, *terrigena*. Inconsistent data published on sequence similarity and DNA-DNA hybridization for some *Haloterrigena* and *Natrinema* strains created some confusion and taxonomic problems initially, but the problems were largely resolved in 2003 by Tindall [4], pointing to uncertainty about strain history. It has been suggested that the discrepancies may also be a result of 16S rDNA interoperon heterogeneity [5]. Published data appears to indicate that both strains GSL-11 and JCM 9743 (formally included in the species *H. turkmenica* by Ventosa *et al*. [1]) may be members of the genus *Natrinema* [4,6]. Those strains will not be considered further here.

There are no reliable reports of other strains of *H. turkmenica* having been isolated. 16S rRNA sequence identity with the other seven type strains in the genus, which were mainly isolated from salt lakes, range from 98.0% for *H. salina* [7] to 94.4% for *H. longa* [6]. The sequence similarity to the *Natrinema* type strains is somewhere in-between, 95.2-96.4% [8], underlining the taxonomic problems [4]. The sequence similarity to phylotypes in environmental metagenomic libraries was not above 87%, indicating a rather poor representation of closely related strains in the habitats analyzed (status January 2010). Here we present a summary classification and a set of features for *H. turkmenica* strain 4k^T^, together with the description of the complete genome sequencing and annotation.

## Classification and features

**Figure 1:**
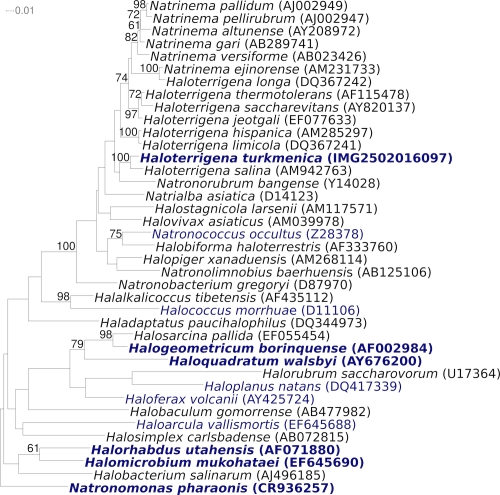
Phylogenetic tree highlighting the position of *H. turkmenica* strain 4k^T^ relative to the other species within the genera *Haloterrigena* and *Natrinema* and the type strains of the other genera within the family *Halobacteriaceae*. The tree was inferred from 1,368 aligned characters [9,10] of the 16S rRNA sequence under the maximum likelihood criterion [11] and rooted with *Natronomonas pharaonis* [12]. The branches are scaled in terms of the expected number of substitutions per site. Numbers above branches are support values from 800 bootstrap replicates [13] if larger than 60%. Strains with a genome sequencing project registered in GOLD [14] are printed in blue; published genomes in bold, *e.g*. the recently published GEBA genomes from *Halogeometricum borinquense* [15], *Halorhabdus utahensis* [16], and *Halomicrobium mukohataei* [17].

Figure 1 shows the phylogenetic neighborhood of *H. turkmenica* strain 4k^T^ in a 16S rRNA based tree. The three 16S rRNA gene sequences in the genome differ from each other by up to two nucleotides, and differ by up to six nucleotides from the previously published 16S rRNA sequence (AB004878) generated from DSM 5511. The difference between the genome data and the previously reported 16S rRNA gene sequences is most likely due to sequencing errors in the previously reported sequence data. As expected, *Haloterrigena* and *Natrinema* strains appear as intermixed in the tree, indicating a paraphyletic status of *Haloterrigena* (within which *Natronorubrum* and *Natrinema* branch off) and of *Natrinema* (within which *H. longa* is placed) [18].

**Figure 2:**
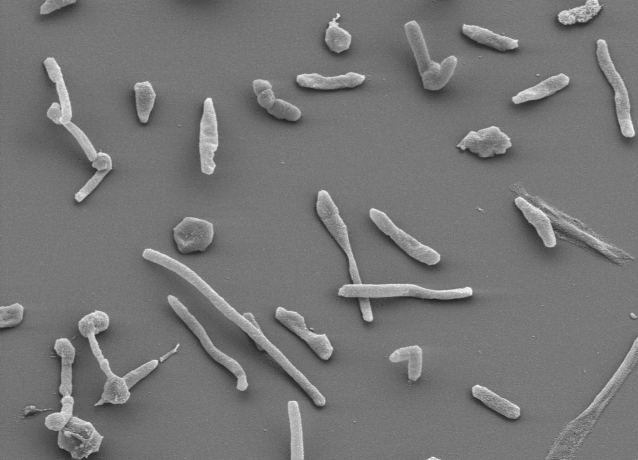
Scanning electron micrograph of *H. turkmenica* strain 4k^T^

*H. turkmenica* cells occur mostly as single cells, rarely in pairs or tetrads [1]. They are described as Gram-negative, ovoid to coccoid, 1.5-2 μm in diameter [1], but can also be rod-shaped (Figure 2 and Table 1) [1]. Neither spores, nor flagella, nor lipid granules were reported. Colonies are pigmented red or light pink due of the presence of C_5O_-carotenoids [1]. Stain 4k^T^ is chemoorganotrophic and aerobic, and requires at least 2 M NaCl [1]. Detailed physiological characteristics were described by Zvyagintseva and Tarasov [3]. The G+C content of DNA was reported to be 59.2-60-2 mol % (Thermal denaturation method [1]), which is significantly less than the 64.3% found in the genome. At optimal growth temperatures, *H. turkmenica* is the fastest growing member of the *Halobacteriaceae*, with only 1.5 hours generation time [26]. Besides the chemical characterization of siderophores [29], there are no published reports on the molecular biology of *H. turkmenica*.

**Table 1:** Classification and general features of *H. turkmenica* 4k^T^ according to the MIGS recommendations [19]

**MIGS ID**	**Property**	**Term**	**Evidence code**
	Current classification	Domain *Archaea*	TAS [20]
		Phylum *Euryarchaeota*	TAS [21,22]
		Class *Halobacteria*	TAS [23]
		Order *Halobacteriales*	TAS [24]
		Family *Halobacteriacea*	TAS [25]
		Genus *Haloterrigena*	TAS [1]
		Species *Haloterrigena turkmenica*	TAS [1]
		Type strain 4k	TAS [3]
	Gram stain	negative	TAS [1]
	Cell shape	rods	TAS [1]
	Motility	nonmotile	IDA
	Sporulation	non-sporulating	NAS
	Temperature range	29-57°C	TAS [26]
	Optimum temperature	51°C	TAS [26]
	Salinity	extreme halophile, requires at least 2% (w/v) NaCl	TAS [1]
MIGS-22	Oxygen requirement	aerobic	TAS [1]
	Carbon source	yeast extract	NAS
	Energy source	chemoorganotroph	TAS [1]
MIGS-6	Habitat	soil	TAS [1]
MIGS-15	Biotic relationship	free living	NAS
MIGS-14	Pathogenicity	none	NAS
	Biosafety level	1	TAS [27]
	Isolation	sulfate saline soil	TAS [3]
MIGS-4	Geographic location	Ashkhabad, Turkmenistan	TAS [3]
MIGS-5	Sample collection time	about or before 1987	TAS [3]
MIGS-4.1 MIGS-4.2	Latitude, Longitude	37.950, 58.380	NAS
MIGS-4.3	Depth	unknown	
MIGS-4.4	Altitude	unknown	

Evidence codes - IDA: Inferred from Direct Assay (first time in publication); TAS: Traceable Author Statement (i.e., a direct report exists in the literature); NAS: Non-traceable Author Statement (i.e., not directly observed for the living, isolated sample, but based on a generally accepted property for the species, or anecdotal evidence). These evidence codes are from of the Gene Ontology project [28]. If the evidence code is IDA, then the property was directly observed by one of the authors or an expert mentioned in the acknowledgements.

Both diphytanyl moieties (C_20_, C_20_) and phytanyl-sesterterpanyl moieties (C_20_, C_25_) are present in polar lipids [1]. The presence of both phytanyl and esterterpanyl side chains implies the presence of three different prenyl transferases involved in lipid biosynthesis, which are probably chain length specific as well as stereospecific for the incorporation of the isoprenoid side chains into the glycerol backbone [30]. The presence of significant levels of both the diphytanyl moieties (C_20_, C_20_) and phytanyl-esterterpanyl moieties (C_20_, C_25_) is characteristic of all members examined of this evolutionary branch of the family *Halobacteriaceae*. Membrane polar lipids are glycerol-diether analogues of PG, PGP-Me and the disulfated digylcosyl diether lipid S_2_-DGD (mannose-2,6 disulfate 1→2 glucose-glycerol diether) [31], the characteristic glycolipid of *Natrialba asiatica* [32]. The presence of respiratory lipoquinones have not been reported, but it may be predicted that MK-8 and MK-8 (VIII-H_2_) should be present, since this is a feature of all members of the family *Halobacteriaceae* examined to date.

## Genome sequencing and annotation information

### Genome project history

This organism was selected for sequencing on the basis of its phylogenetic position, and is part of the *** G****enomic* *** E****ncyclopedia of* *** B****acteria and* *** A****rchaea * project [33]. The genome project is deposited in the Genomes OnLine Database [14] and the complete genome sequence in GenBank. Sequencing, finishing and annotation were performed by the DOE Joint Genome Institute (JGI). A summary of the project information is shown in Table 2.

**Table 2:** Genome sequencing project information

**MIGS ID**	**Property**	**Term**
MIGS-31	Finishing quality	Finished
MIGS-28	Libraries used	Three genomic libraries: one Sanger 8 kb pMCL200 library, one 454 pyrosequence standard library and one Illumina standard library
MIGS-29	Sequencing platforms	ABI3730, 454 GS FLX, and Illumina GA
MIGS-31.2	Sequencing coverage	6.9× Sanger; 19.9× pyrosequence
MIGS-30	Assemblers	Newbler version 1.1.03.24, phrap
MIGS-32	Gene calling method	Prodigal 1.4, GenePRIMP
	Genbank ID	CP001860 (chromosome) CP001861-CP001866 (plasmids)
	Genbank Date of Release	January 19, 2010
	GOLD ID	Gc01189
	NCBI project ID	30411
	Database: IMG-GEBA	2501939622
MIGS-13	Source material identifier	DSM 5511
	Project relevance	Tree of Life, GEBA

### Growth conditions and DNA isolation

*H. turkmenica* 4k^T^, DSM 5511, was grown in DSMZ medium 372 (*Halobacteria* medium) [34] at 37°C. DNA was isolated from 1-1.5 g of cell paste using Qiagen Genomic 500 DNA Kit (Qiagen, Hilden, Germany) with lysis modification L according to Wu *et al*. [33].

### Genome sequencing and assembly

The genome was sequenced using a combination of Sanger and 454 sequencing platforms. All general aspects of library construction and sequencing performed at the JGI can be found at the JGI website (http://www.jgi.doe.gov/). 454 Pyrosequencing reads were assembled using the Newbler assembler version 1.1.03.24 (Roche). Large Newbler contigs were broken into 6,060 overlapping fragments of 1,000 bp and entered into assembly as pseudo-reads. The sequences were assigned quality scores based on Newbler consensus q-scores with modifications to account for overlap redundancy and adjust inflated q-scores. A hybrid 454/Sanger assembly was made using the parallel phrap assembler (High Performance Software, LLC). Possible misassemblies were corrected with Dupfinisher or transposon bombing of bridging clones [35]. A total of 1,183 Sanger finishing reads were produced to close gaps, to resolve repetitive regions, and to raise the quality of the finished sequence. Illumina reads were used to improve the final consensus quality using an in-house developed tool (the Polisher). The error rate of the completed genome sequence is less than 1 in 100,000. Together, the combination of the Sanger and 454 sequencing platforms provided 26.8× coverage of the genome. The final assembly contains 33,433 Sanger reads and 394,632 pyrosequencing reads.

### Genome annotation

Genes were identified using Prodigal [36] as part of the Oak Ridge National Laboratory genome annotation pipeline, followed by a round of manual curation using the JGI GenePRIMP pipeline [37]. The predicted CDSs were translated and used to search the National Center for Biotechnology Information (NCBI) nonredundant database, UniProt, TIGR-Fam, Pfam, PRIAM, KEGG, COG, and InterPro databases. Additional gene prediction analysis and functional annotation was performed within the Integrated Microbial Genomes - Expert Review (http://img.jgi.doe.gov/er) platform [38].

### Genome properties

**Figure 3:**
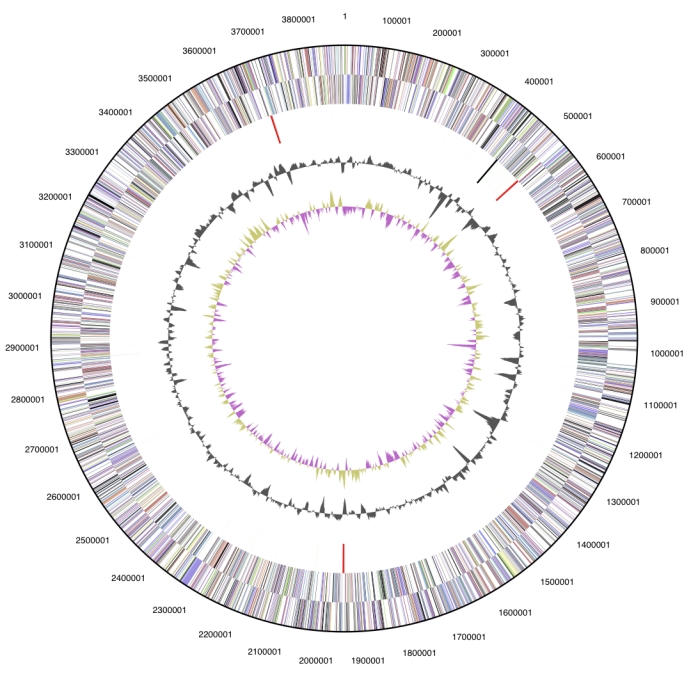
Graphical circular map of the chromosome. From outside to the center: Genes on forward strand (color by COG categories), Genes on reverse strand (color by COG categories), RNA genes (tRNAs green, rRNAs red, other RNAs black), GC content, GC skew.

**Figure 4:**
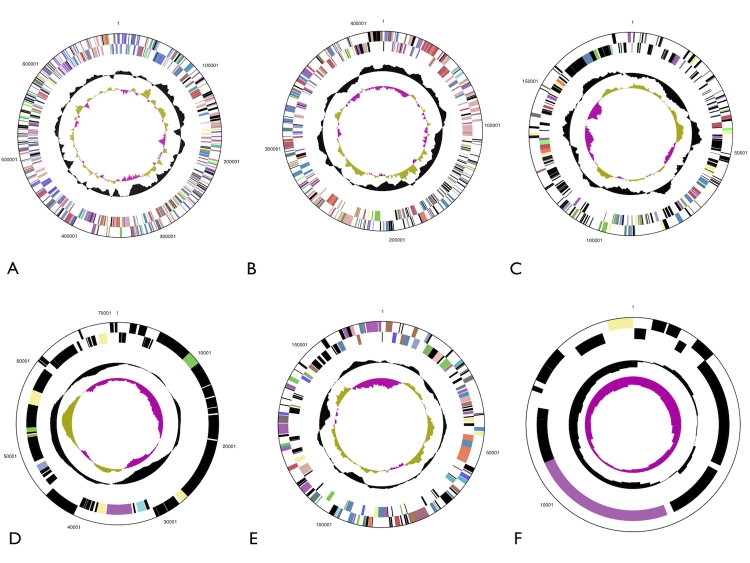
Graphical circular map of the six plasmids: pHTUR01 (A), pHTUR02 (B), pHTUR03 (C), pHTUR04 (D), pHTUR05 (E), pHTUR06 (F)**.** Plasmids not drawn to scale**.**

The genome is 5,440,782 bp long and comprises one main circular chromosome of 3,889,038 bp length and six circular plasmids of 15.8 to 698.5 kbp length, with an overall GC content of 64.3% (Table 3 and Figures 3 and 4). Of the 5,350 genes predicted, 5,287 were protein coding genes, and 63 RNAs; 174 pseudogenes were also identified. The majority of the protein-coding genes (60.1%) were assigned a putative function while those remaining were annotated as hypothetical proteins. The distribution of genes into COGs functional categories is presented in Table 4.

**Table 3:** Genome Statistics

**Attribute**	Value	% of Total
Genome size (bp)	5,440,782	100.00%
DNA coding region (bp)	4,524,412	83.16%
DNA G+C content (bp)	3,496,479	64.26%
Number of replicons	7	
Extrachromosomal elements	6	
Total genes	5,350	100.00%
RNA genes	63	1.18%
rRNA operons	3	
Protein-coding genes	5,287	98.82%
Pseudo genes	174	3.25%
Genes with function prediction	3,213	60.06%
Genes in paralog clusters	1,706	31.89%
Genes assigned to COGs	3,259	60.92%
Genes assigned Pfam domains	3,208	59.96%
Genes with signal peptides	625	11.68%
Genes with transmembrane helices	1,140	21.31%
CRISPR repeats	1	

**Table 4:** Number of genes associated with the general COG functional categories

**Code**	**Value**	**%age**	**Description**
J	178	3.4	Translation, ribosomal structure and biogenesis
A	1	0.0	RNA processing and modification
K	190	3.6	Transcription
L	150	2.8	Replication, recombination and repair
B	3	0.1	Chromatin structure and dynamics
D	35	0.7	Cell cycle control, mitosis and meiosis
Y	0	0.0	Nuclear structure
V	44	0.8	Defense mechanisms
T	161	3.0	Signal transduction mechanisms
M	125	2.4	Cell wall/membrane biogenesis
N	29	0.5	Cell motility
Z	0	0.0	Cytoskeleton
W	0	0.0	Extracellular structures
U	26	0.5	Intracellular trafficking and secretion
O	141	2.7	Posttranslational modification, protein turnover, chaperones
C	258	4.9	Energy production and conversion
G	221	4.2	Carbohydrate transport and metabolism
E	349	6.6	Amino acid transport and metabolism
F	78	1.5	Nucleotide transport and metabolism
H	189	3.6	Coenzyme transport and metabolism
I	176	3.3	Lipid transport and metabolism
P	224	4.2	Inorganic ion transport and metabolism
Q	87	1.6	Secondary metabolites biosynthesis, transport and catabolism
R	630	11.9	General function prediction only
S	321	6.1	Function unknown
-	2,091	39.5	Not in COGs
